# Development of Artificial System to Induce Chromatin Loosening in *Saccharomyces cerevisiae*

**DOI:** 10.3390/biom12081138

**Published:** 2022-08-18

**Authors:** Ryota Yamamoto, Genki Sato, Takamitsu Amai, Mitsuyoshi Ueda, Kouichi Kuroda

**Affiliations:** Division of Applied Life Sciences, Graduate School of Agriculture, Kyoto University, Sakyo-ku, Kyoto 606-8502, Japan

**Keywords:** chromatin, histone acetyltransferase, epigenetics, CRISPR/dCas9 system, *Saccharomyces cerevisiae*

## Abstract

In eukaryotic cells, loosening of chromatin causes changes in transcription and DNA replication. The artificial conversion of tightly packed chromatin (heterochromatin) to loosely packed chromatin (euchromatin) enables gene expression and regulates cell differentiation. Although some chemicals convert chromatin structures through histone modifications, they lack sequence specificity. This study attempted to establish a novel technology for inducing chromatin loosening in target regions of *Saccharomyces cerevisiae*. We focused on histone acetylation, which is one of the mechanisms of euchromatin induction. The sequence-recognizing ability of the dead Cas9 (dCas9) and guide RNA (gRNA) complex was used to promote histone acetylation at a targeted genomic locus. We constructed a plasmid to produce a fusion protein consisting of dCas9 and histone acetyltransferase Gcn5 and a plasmid to express gRNA recognizing the upstream region of heterochromatic *URA3*. Confocal microscopy revealed that the fusion proteins were localized in the nucleus. The yeast strain producing the fusion protein and gRNA grew well in the uracil-deficient medium, while the strain harboring empty plasmids or the strain containing the mutations that cause loss of nucleosomal histone acetylation activity of Gcn5 did not. This suggests that the heterochromatin was loosened as much as euchromatin through nucleosomal histone acetylation. The amount of euchromatic DNA at the target locus increased, indicating that chromatin loosening was induced by our system. Nucleosomal histone acetylation in heterochromatic loci by our developed system is a promising method for inducing euchromatic state in a target locus.

## 1. Introduction

Epigenetics is the study of molecules and mechanisms that perpetuate alternative gene-activated states in the context of the same DNA sequence [1,2]. Typical epigenetic phenomena include X-chromosome inactivation [3], genome imprinting [4], variation of position effect [5], and cell differentiation [6,7]. Chromatin condensation, another epigenetic phenomenon, affects the accessibility of proteins required for gene transcription, DNA replication, recombination, and repair [8,9,10]. Chromatin states are classified as condensed heterochromatin or loosened euchromatin [11]. In the euchromatic region, transcription is activated because transcription factors can easily access the DNA. In contrast, transcription is inactivated in the heterochromatic region because these elements are inaccessible to DNA [8,12]. Therefore, the reversible transition of the chromatin state can be used to regulate gene expression upstream of regulation using DNA and transcription factors [9,10], and the establishment of techniques to regulate the chromatin state would enable the comprehensive and reversible control of gene expression. Changes in chromatin state result in the regulation of cellular differentiation [13]. Furthermore, chromatin abnormalities are known to cause diseases [14]. Thus, a technology to regulate the level of chromatin condensation may have applicability in the medical field.

However, there have been few reports of artificial control of chromatin condensation levels. Currently, the most common method for regulating chromatin state is the use of chemical catalysts [15]. This can be used for the acetylation of histone proteins. However, chemical catalysis modifies histone proteins in a wide range of genomic loci in a non-specific manner. Therefore, it is difficult to loosen the partial heterochromatin at a specific locus. Moreover, some examples of epigenetic regulation of gene expression using clustered regularly interspaced short palindromic repeat (CRISPR) technologies have recently been reported [16]. In genome editing using CRISPR/Cas9, the Cas9 and guide RNA (gRNA), in which the 3′ end of crRNA is fused to the 5′ end of tracrRNA, are used [17]. A complex of Cas9 and gRNA is directed to its target DNA according to the complementarity of the 20 nucleotides at the 5′ end of the gRNA [17]. After the complex recognizes the target sequence, double-strand breaks (DSBs) are induced in three base pairs upstream of PAM [17]. However, dead Cas9 (dCas9), which lacks nuclease activity by inducing two-point mutations (D10A and H840A) within the Cas9 gene, forms a complex with gRNA in the target DNA region but does not form DSBs [18]. Therefore, dCas9 can be used to carry the desired enzymes to the target DNA region without cutting the target DNA (CRISPR/dCas9 system). There are some examples of altering gene expression using CRISPR/dCas9 systems, including the fusion of transcriptional activators [19,20], histone modification enzymes [21,22,23], and DNA methylation enzymes with dCas9 [24,25]. However, it has not been confirmed that epigenome editing using histone-modifying enzymes converts the chromatin condensation state [21,22,23]. In addition, epigenome editing using these technologies was not performed in heterochromatic regions [21,22,23]. Therefore, it is necessary to develop a technology to loosen a target locus in the heterochromatic regions.

In this study, we attempted to establish a technology to induce transitions to euchromatin in a targeted locus. The mechanism of the transition between euchromatin and heterochromatin has been well understood. It has been proposed that histone acetylation and a specific type of methylation lead to euchromatin, whereas deacetylation and a different type of methylation lead to heterochromatin [11]. Among these, the action of histone acetylases is important for the transition to the euchromatic state [11]. It has been suggested that electrostatic binding between acidic DNA and basic histone proteins is weakened by acetylation, which neutralizes the charge on lysine residues, thus favoring transcriptional activation [12]. Therefore, we introduced histone acetylation of a target locus in *S. cerevisiae* to induce a euchromatic state using histone acetyltransferases (HATs) that acetylate lysine of histones. HATs are classified as type A and type B based on the differences in acetylated histones [26]. Type A acetylates histones that constitute nucleosomes in the nucleus, and type B acetylates newly synthesized histones in the cytoplasm [26]. It is also divided into the Gcn5-related *N*-acetyltransferase (GNAT) family and the MYST (MOZ, Ybf2/Sas3, Sas2, and Tip60) family based on the difference in the reaction mode. In addition, it has been reported that HAT acts as a transcriptional coactivator, such as CBP/p 300 in humans [26]. Gcn5 was used to induce histone acetylation at the target locus. Gcn5 is a histone acetyltransferase classified in the GNAT family, which mainly acetylates lysine in histone H3 proteins [27,28] and functions as a coactivator of transcription [27,29].

To establish a chromatin-loosening technique in a target heterochromatic locus in yeast, we fused the histone acetyltransferase Gcn5 from yeast to dCas9 and constantly expressed it together with a guide RNA (gRNA) that recognizes a heterochromatic target locus to be loosened. Using our system, we induced *URA3* expression in the heterochromatic locus by the dCas9-Gcn5 fusion protein in the nucleosomal histone acetylation activity-dependent manner and obtained yeast cells with loosened chromatin in this target locus. To our knowledge, this study is the first to loosen a target heterochromatic locus using dCas9-Gcn5 that has the nucleosomal histone acetylation activity. In addition, we showed that histone acetylation in heterochromatic loci by dCas9-Gcn5 and gRNA is a promising method for inducing a euchromatic state in a target locus.

## 2. Materials and Methods

### 2.1. Strains, Media, and Culture Conditions

*Escherichia coli* strain DH5α [F^−^, Φ80d*lacZ*ΔM15, Δ(*lacZYA*-*argF*)U169, *deoR*, *recA1*, *endA*1, *hsdR*17 (r_K_^−^, m_K_^+^), *phoA*, *supE*44, λ^−^, *thi-*1, *gyrA*96, *relA*1] (Toyobo, Osaka, Japan) was used as the host for recombinant DNA manipulation. DH5α cells were grown in LB + A medium (1% (*w*/*v*) tryptone (Becton, Dickinson and Company, Franklin Lakes, NJ, USA), 0.5% (*w*/*v*) yeast extract (Becton, Dickinson and Company), and 0.5% (*w*/*v*) sodium chloride (Nacalai tesque, Kyoto, Japan)) containing 100 μg/mL ampicillin (Meiji Seika Pharma CO., Tokyo, Japan). The genotypes of the *S. cerevisiae* W303-1A (ATCC, Manassas, VA, USA), UCC3505 (ATCC), and UCC3505 derivatives, whose *ura3* mutation was recovered and/or *ADA2* was deleted, are listed in Table S1. These yeast strains were cultured in the following medium: YPD (1% (*w*/*v*) yeast extract (Becton, Dickinson and Company), 2% (*w*/*v*) peptone (Becton, Dickinson and Company), and 2% (*w*/*v*) glucose (Nacalai tesque)); YPD + G418 (1% (*w*/*v*) yeast extract, 2% (*w*/*v*) peptone, 2% (*w*/*v*) glucose, and 300 μg/mL G418 (Nacalai tesque)); SDC + AU (0.67% (*w*/*v*) yeast nitrogen base without amino acids (Becton, Dickinson and Company), 2% (*w*/*v*) glucose, 0.5% (*w*/*v*) casamino acids (Becton, Dickinson and Company), 0.002% (*w*/*v*) adenine (Nacalai tesque), and 0.002% (*w*/*v*) uracil (Nacalai tesque)); SDC + U (0.67% (*w*/*v*) yeast nitrogen base without amino acids, 2% (*w*/*v*) glucose, 0.5% (*w*/*v*) casamino acids, and 0.002% (*w*/*v*) uracil); SDC + 5 × U (0.67% (*w*/*v*) yeast nitrogen base without amino acids, 2% (*w*/*v*) glucose, 0.5% (*w*/*v*) casamino acids, and 0.01% (*w*/*v*) uracil); SDC (0.67% (*w*/*v*) yeast nitrogen base without amino acids, 2% (*w*/*v*) glucose, 0.5% (*w*/*v*) casamino acids); SDC + AWU (0.67% (*w*/*v*) yeast nitrogen base without amino acids, 2% (*w*/*v*) glucose, 0.5% (*w*/*v*) casamino acids, 0.002% (*w*/*v*) adenine, 0.002% (*w*/*v*) tryptophan (Nacalai tesque), and 0.002% (*w*/*v*) uracil); and SDC + AW (0.67% (*w*/*v*) yeast nitrogen base without amino acids, 2% (*w*/*v*) glucose, 0.5% (*w*/*v*) casamino acids, 0.002% (*w*/*v*) adenine, and 0.002% (*w*/*v*) tryptophan).

### 2.2. Construction of Expression Plasmids

All the primers used for plasmid construction are listed in Table S2, and all the plasmids used in this study are listed in Table S3. All DNA fragments were amplified via polymerase chain reaction (PCR) using KOD-One DNA polymerase (Toyobo). The open reading frame of *GCN5* was amplified using primers (pUC19_Gcn5_Fw and pUC19_Gcn5_Rv) from *S. cerevisiae* W303-1A genomic DNA. *S. cerevisiae* W303-1A genomic DNA was extracted from yeast cells cultured in YPD medium using Dr. GenTLE^TM^ High Recovery (Takara Bio, Shiga, Japan) according to the manufacturer’s instructions. The amplified DNA fragment was cloned into pUC19 [30], which was predigested with BamHI (Toyobo), using an In-Fusion HD Cloning kit (Takara Bio) according to the manufacturer’s instructions. The resulting plasmid was named pUC19_Gcn5. The dCas9-encoding sequence was obtained by introducing two-point mutations (D10A and H840A) into the Cas9 gene from p414-TEF1p-Cas9-CYC1t [31] which encodes Cas9 from *Streptococcus pyogenes*, using the QuickChange method with primers QC Cas9 D10A_Fw, QC Cas9 D10A_Rv, QC Cas9 H840A_Fw, and QC Cas9 H840A_Rv. The resulting plasmid was named pdCas9. The nuclear localization signal (NLS) of the SV40 large T antigen [32] was used in this study. Linker-encoding sequences were also inserted between the dCas9- and Gcn5-encoding sequences. The sequences encoding the NLSs and linkers are listed in Table S4. The DNA fragment encoding NLS-dCas9 was amplified from pdCas9 using primers containing NLS- or linker-encoding sequences (pWGP3_NLS-dCas9_Fw and pWGP3_dCas9-linker_Rv, respectively). The *GCN5* ORF was amplified from pUC19_Gcn5 using primers containing a linker-encoding sequence (Linker-Gcn5_Fw and Gcn5-EGFP_Rv). The DNA fragment encoding enhanced green fluorescent protein (EGFP) was amplified from pEGFP (Clontech, Mountain View, CA, USA) using primers EGFP_Fw and pWGP3-EGFP_Rv. A multicopy plasmid, pWGP3 [33], was used to constitutively express the NLS-dCas9-Gcn5 fusion protein. All amplified DNA fragments (NLS-dCas9-, linker-, Gcn5-, and EGFP-encoding sequences) were inserted downstream of the *GAPDH* promoter in pWGP3, which was predigested with BamHI (Toyobo). The resulting plasmid was named pWGP3_NLS-dCas9-Gcn5-EGFP. In addition, the DNA fragment encoding NLS-dCas9 or Gcn5 was amplified using primers including NLS-or linker-encoding sequences (pWGP3_NLS-dCas9_Fw and pWGP3_dCas9-linker_Rv) or (Linker-Gcn5_Fw and pWGP3_Gcn5_Rv), respectively. These DNA fragments (NLS-dCas9- and linker-Gcn5-encoding sequences) were inserted into pWGP3, as described above. The resulting plasmid was named pWGP3_NLS-dCas9-Gcn5. 

A centromeric plasmid, p414 GPD [34], was used to constitutively express the NLS-dCas9-Gcn5 fusion. The NLS-dCas9-encoding sequence was amplified by PCR using primers containing the NLS-encoding sequences (p414G_NLS_Fw and p414G_dCas9_Rv) from pWGP3_NLS-dCas9-Gcn5-EGFP. The amplified DNA fragment was then inserted into p414 GPD, which was predigested with Spe I (Toyobo). The resulting plasmid was named p414 GPD_NLS-dCas9. The Gcn5-EGFP-encoding sequence was amplified from pWGP3_NLS-dCas9-Gcn5-EGFP using the primers p414G_dCas9EcoRI_Fw and p414G_EGFP_Rv. The amplified DNA fragment was inserted into p414 GPD_NLS-dCas9, which was predigested with EcoRI (Toyobo). The resulting plasmid was named p414 GPD_NLS-dCas9-Gcn5-EGFP. The *GCN5* ORF was also amplified from pWGP3_NLS-dCas9-Gcn5 using the primers p414G_dCas9EcoRI_Fw and p414G_Gcn5_Rv and inserted into p414 GPD_NLS-dCas9, which was predigested with EcoRI (Toyobo). The resulting plasmid was named p414 GPD_NLS-dCas9-Gcn5. To construct the Gcn5^KQL126–128AAA^ mutant [35], the 3′-terminal 304 bp of dCas9 ORF, linker, and 5′-terminal 384 bp of *GCN5* ORF including KQL126-128AAA mutation was also amplified from p414_GPD_NLS-dCas9-Gcn5 using the primers p414G_dCas9EcoRI_Fw and GCN5_KQLMt_Rv, and 3′-terminal 956 bp of *GCN5* ORF with the same mutation was also amplified from p414_GPD_NLS-dCas9-Gcn5 using the primers GCN5_KQLMt_Fw and p414G_Gcn5_Rv. Then, these fragments were inserted into p414 GPD_NLS-dCas9, which was predigested with EcoRI (Toyobo). The resulting plasmid was named p414 GPD_NLS-dCas9-Gcn5 ^KQL126–128AAA^. All amplified DNA fragments were inserted into the backbone plasmids using an In-Fusion HD Cloning kit (Takara Bio) according to the manufacturer’s instructions. 

The plasmid for expressing gRNA was constructed by replacing the gRNA-targeting sequence with a marker gene in 426_gRNA-empty [36]. *ADE2*, an auxotrophic marker gene, was amplified from pRS402 [37] using the primers Ade2_Fw and Ade2_Rv. The amplified *ADE2* sequence was inserted into the amplified plasmid from p426_gRNA-empty using the primers p426_Fw and p426_Rv by an In-Fusion HD Cloning kit (Takara Bio) according to the manufacturer’s instructions. The resulting plasmid was named p422_gRNA-empty plasmid. The DNA fragment encoding gRNA, which recognizes the upstream region of heterochromatic *URA3* inserted in the telomeric region of *S. cerevisiae* UCC3505, was prepared by incubating the primers (hUra3_Fw and hUra3_Rv) at 90 °C for 10 min and slowly cooling to 4 °C. This DNA fragment was ligated into the plasmid p422_gRNA-empty digested with BsaI (New England BioLabs, Ipswich, MA, USA) using Ligation High Ver. 2 (Toyobo) according to the manufacturer’s instructions. The resulting plasmid was named p422_gRNA-hUra3. For the cell growth assay, an empty plasmid, p422 GPD, was constructed by replacing the marker gene (*URA3*) in p426 GPD with *ADE2*. Backbone sequences were amplified by PCR using the primers p426_Fw and p426_Rv from p426 GPD. The *ADE2* sequence was amplified from pRS402 using the primers (Ade2_Fw and Ade2_Rv). The amplified *ADE2* gene was then inserted into the backbone by TEDA cloning [38] and transformed into *E. coli*. 

Plasmids sequences were confirmed using Sanger sequencing using primers (pUC19_seq_Fw, pUC19_seq_Rv, GAPDH promoter_Fw, CYC terminator_Rv, dCas9_1_Fw, dCas9_2_Fw, dCas9_3_Fw, dCas9_4_Fw, dCas9_5_Fw, dCas9_6_Fw, Gcn5_1_Fw, and Gcn5_2_Fw).

### 2.3. Yeast Transformation

The constructed plasmids were introduced into yeast using the Frozen-EZ Transformation kit II (Zymo Research, Irvine, CA, USA) according to the manufacturer’s instructions. Transformants were selected on SDC + AU agar plates or SDC + U liquid media.

### 2.4. Recovery of ura3 Mutation and Deletion of ADA2 in S. cerevisiae UCC3505

All primers used for reversion of the *ura3* mutation and deletion of *ADA2* in *S. cerevisiae* UCC3505 are listed in Table S2. The DNA fragments of the upstream and downstream sequences of *URA3* were amplified by PCR from *S. cerevisiae* UCC3505 genomic DNA using the primer pairs Ura3_up_Fw and Ura3_up_Rv and Ura3_down_Fw and Ura3_down_Rv, respectively. KOD FX Neo DNA polymerase (Toyobo) was used to amplify DNA fragments. In addition, *S. cerevisiae* UCC3505 genomic DNA was extracted from yeast cells cultured in YPD medium using Dr. GenTLE^TM^ High Recovery (Takara Bio) according to the manufacturer’s instructions. *URA3* was amplified from p416 GPD using the primers URA3_Fw and URA3_Rv. The amplified DNA fragments (upstream sequence of *URA3*, ORF of *URA3*, and downstream sequence of *URA3*) were inserted into pUC19 predigested with BamHI (Toyobo) using an In-Fusion HD Cloning kit (Takara Bio) according to the manufacturer’s instructions. The resulting plasmid was named pUC19-URA3. The inserted DNA fragment for the reversion of the *ura3* mutation was amplified from pUC19-URA3 recovery using KOD-One DNA polymerase (Toyobo) and the primers URA3_up_Fw and URA3_down_Rv. The amplified DNA fragment was purified using the FastGene Gel/PCR Extraction kit (NIPPON Genetics, Tokyo, Japan) and introduced into *S. cerevisiae* UCC3505 using an EZ-yeast Transformation kit (Zymo Research) according to the manufacturer’s instructions. The transformants were selected on SDC + AW agar plates. Single colonies that formed on SDC + AW agar plates were isolated. Yeast genomic DNA was extracted by incubating the yeast cells in 5 mM NaOH at 90 °C for 10 min and slowly cooling to 4 °C. Genomic *URA3* sequences were amplified from the extracted genomic DNA via PCR using KOD FX Neo DNA polymerase (Toyobo) and the primers Ura3_coloP_Fw and Ura3_coloP_Rv, and its sequence was confirmed by Sanger sequencing using the primers Ura3_seq_Fw and Ura3_seq_Rv.

The *ADA2* was deleted following the above methods. Briefly, the DNA fragment consisting of the 5′ flanking sequence of the *ADA2* ORF, the *kanMX4* gene, and the 3′ flanking sequence of the *ADA2* ORF was amplified from the genomic DNA of the BY4741 *ada2*Δ strain (Euroscarf, Oberursel, Germany) using the primers ADA2_A_L and ADA2_D_L. The amplified DNA fragment was introduced into UCC3505 and UCC3505 *URA3* recovery strains and the transformants were selected on YPD + G418 plates. The *ADA2* deletion of each single colony was confirmed by colony direct PCR using the primer sets (ADA2_A and ADA2_D) and (ADA2_D and KanC). The genomic sequences including the ADA2 locus were amplified using the primers ADA2_A and ADA2_D, and confirmed by Sanger sequencing using the primers KanC, KanB, and KanB_2.

### 2.5. Fluorescence Microscopy

For fluorescence microscopy, transformed yeast cells were cultured in SDC + AU at 30 °C. Cell cultures were collected when the optical density at 600 nm (OD_600_) reached 0.4. Twenty microliters of 1 mg/mL DAPI solution (Thermo Fisher Scientific, Waltham, MA, USA) was added to 1 mL of yeast cell culture. The DAPI-added cell culture was then incubated at 30 °C for 30 min and washed twice with PBS (pH 7.4). Cells were observed using confocal laser scanning microscopy (LSM700; Carl Zeiss, Oberkochen, Germany) with a 100× objective lens (oil immersion numerical aperture (NA), 1.40). EGFP and DAPI were detected at 488 and 405 nm excitation, respectively. The fluorescent emission of EGFP was measured between 490 and 550 nm and that of DAPI was measured at 550 nm. The acquired images were processed using the ZEN 2011 SP7 FP1 software (Carl Zeiss, black edition, version 14.0.8.201) and edited with Microsoft PowerPoint (Microsoft, Redmond, WA, USA, version 2207).

### 2.6. Cell Growth Assay

Competent cells of each yeast strain were prepared by EZ-yeast Transformation kit (Zymo Research, Irvine, CA, USA) according to the manufacturer’s instructions, and their transformation efficiency was assessed for each introduced plasmid. Equal amounts of yeast transformants that were adjusted according to the transformation efficiency were directly suspended in 4 mL SDC+ 5 × U liquid medium and cultured at 250 rpm and 30 °C for 48 h. The harvested cells were washed with 4 mL of PBS (10 mM Na_2_HPO_4_, 1.76 mM NaH_2_PO_4_, 137 mM NaCl, and 2.7 mM KCl, pH 7.4) twice and then diluted to 0.2 of OD_600_ in 200 μL SDC liquid medium on the 96-well Polystyrene Microplates (353072; Corning, Corning, NY, USA). During the incubation at 30 °C without shaking, the cell growth curve was monitored by measuring absorbance at 600 nm of cell culture with the plate reader (Tecan Infinite 200 Pro F Plex; Tecan Ltd., Männedorf, Switzerland). The plates were sealed tightly by the transparent sealing tape (232698; Thermo Fisher Scientific), and the moat was filled by 70 μL of sterilized 0.1% agarose (Nacalai tesque) to reduce evaporation of the medium. The maximum specific growth rate was calculated by using Curveball 0.2.16 [39].

### 2.7. Cell Growth Assay in S. cerevisiae for Analyzing Plasmid Loss

Yeast strains expressing dCas9-Gcn5 fusion protein and gRNA were cultured in YPD liquid medium at 30 °C for 48 h. Harvested cells were streaked onto YPD plates and incubated at 30 °C. Single colonies were streaked on SDC + U plates, incubated at 30 °C for 2 days, and plasmid retention was determined from colony formation. Yeast strains that had lost their plasmid were streaked on YPD plate, incubated in SDC + AWU liquid medium at 30 °C overnight, and stored in 15% glycerol stocks. Yeast cells from glycerol stocks were streaked onto SDC + AWU plates. After incubation at 30 °C for 2 days, single colonies were cultured in SDC + AWU liquid medium at 250 rpm and 30 °C for 48 h. The harvested cells were washed with 400 μL of SDC + AW liquid medium twice and then diluted to 0.1 of OD_600_ in SDC + AW liquid medium. During the incubation with constant shaking at 250 rpm and 30 °C, the cell growth was evaluated by measuring OD_600_ of cell culture.

### 2.8. Formaldehyde-Assisted Isolation of Regulatory Elements (FAIRE)-qPCR Assay

Yeast transformants were cultured in SDC + U liquid medium at 30 °C for 48 h. The harvested cells were washed with SDC liquid medium and then diluted to an OD_600_ of 0.1 in SDC liquid medium. After incubating at 250 rpm and 30 °C for 2 days, 100 μL of cell culture was spread on an SDC plate and incubated at 30 °C. Single colonies were inoculated into 10 mL of SDC + U liquid medium, incubated at 250 rpm and 30 °C overnight, and stored in 15% glycerol stocks. Yeast cells from glycerol stocks were streaked onto SDC + U plates. After incubation at 30 °C for 2 days, single colonies were cultured in SDC + U liquid medium overnight at 250 rpm and 30 °C. Harvested cells were used for the evaluation of their growth following the aforementioned growth assay methods. For FAIRE-qPCR assay, single colonies were cultured in SDC + U liquid medium at 250 rpm and 30 °C for 16 h until OD_600_ reached 5.0. Yeast cells were fixed by adding formaldehyde at a final concentration of 1% and incubating for 15 min at 30 °C with shaking at 250 rpm. The cross-linking reaction was quenched by adding glycine to a final concentration of 125 mM and incubating for 5 min at 250 rpm and 30 °C. After the cells were collected by centrifugation for 5 min at 1500× *g* and 4 °C, the cell pellets were washed twice with 1 mL of cold PBS (pH 7.4). The cell pellets were then resuspended in 500 µL of lysis buffer (10 mM Tris–HCl pH 8.0, 1 mM EDTA, 100 mM NaCl, 2% Triton X-100, and 1% SDS). After adding 5 µL of Halt Protease Inhibitor Cocktail (Thermo Fisher Scientific) and 5 µL of PMSF (ACTIVE MOTIF, Carlsbad, CA, USA), the cell suspensions were transferred to 2.0 mL screw-cap tubes. Then, 0.5 mm glass beads (TOMY, Tokyo, Japan) were added, and the cells were disrupted using the Bead Smash 12 bead beater (Wakenyaku, Kyoto, Japan) with five cycles of 30 s shaking at 4000 rpm and 30 s on ice with 30 s intervals on ice between cycles. The disrupted cells were centrifuged for 2 min at 400× *g* and 4 °C, and the supernatant was collected. Chromatin in the collected supernatant was sheared in a Bioruptor UCD-250 (Cosmo Bio, Tokyo, Japan) with 30 cycles of 10 s at 200 W output and 30 s off on ice to generate fragments of 300–400 bp and then divided into two aliquots for further experiments as input or FAIRE samples. Fragment sizes were confirmed using an Agilent 2100 bioanalyzer (Agilent Technologies, Santa Clara, CA, USA) and an Agilent High Sensitivity DNA kit (Agilent Technologies) according to the manufacturer’s instructions.

Chromatin in the input DNA samples was de-cross-linked by adding RNase A (ACTIVE MOTIF) at a final concentration of 0.1 mg/mL and incubating at 37 °C for 60 min, followed by the addition of proteinase K (Nacalai tesque) at a final concentration of 0.2 mg/mL and overnight incubation at 65 °C. The input DNA samples as well as the FAIRE DNA samples were purified with three rounds of phenol-chloroform extraction followed by ethanol precipitation. Chromatin in the purified FAIRE samples was de-cross-linked with RNase A, as described above. Finally, the extracted DNA fragments in FAIRE and input DNA samples were purified using the QIAquick PCR and Gel Cleanup kit (Qiagen, Hilden, Germany) according to the manufacturer’s instructions.

The relative amounts of euchromatic DNA in the target region in the FAIRE and input DNA samples were determined by qPCR. All the primers used for FAIRE-qPCR are listed in Table S2. The PCR solution was prepared using FastStart SYBR Green Master (Roche, Mannheim, Germany) and dispensed into each well of a 96-well plate. The *GPD* promoter region was used as the euchromatic DNA region for this analysis. The *adh4::URA3* region was used to measure the amount of euchromatic DNA present in the target region. The calculated relative amount of euchromatic DNA was normalized to the amount of the input DNA samples. The *GPD* promoter was amplified using the primers GPD_qPCR_Fw and GPD_qPCR_Rv. The *adh4::URA3* region was amplified using the primers (hUra3_qPCR_Fw and hUra3_qPCR_Rv). Fluorescence was detected using a C1000 thermal cycler (Bio-Rad Laboratories, Hercules, CA, USA) and a CFX96 real-time system (Bio-Rad Laboratories). Data were acquired and analyzed using the CFX manager software (Bio-Rad Laboratories, version 2.1.1022.0523.) and Microsoft Excel (Microsoft, version 2207). The specificity of the primer sets used for qPCR was confirmed by confirming the sequence of qPCR products with Sanger sequencing. 

### 2.9. Statistical Analysis

Error bars represent the SEM of three independent experiments. Statistical significance was evaluated by a two-tailed Student’s *t*-test and the Holm method was used to control the family-wise error rates. Statistical details for each experiment can be found in figure legends. *p*-values < 0.05 were considered statistically significant.

## 3. Results

### 3.1. Nuclear Localization of the dCas9-Gcn5 Fusion Protein

**Figure 1:**
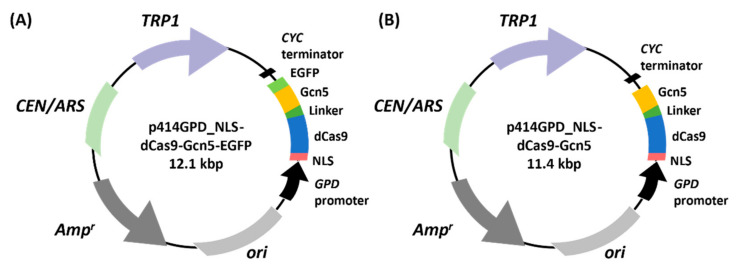
Construction of plasmids for producing fusion protein of dCas9, Gcn5, and EGFP in the nucleus. NLS-dCas9-Gcn5-EGFP or NLS-dCas9-Gcn5 was constitutively produced under the control of the *GPD* promoter by introducing the centromeric plasmid p414 GPD_NLS-dCas9-Gcn5-EGFP (**A**) or p414 GPD_NLS-dCas9-Gcn5 (**B**), respectively. NLS, nuclear localization signal; dCas9, dead Cas9; EGFP, enhanced GFP.

To establish a technology to induce chromatin loosening in a targeted locus, we designed a fusion protein consisting of dCas9 for recruitment to a target locus and Gcn5 for histone acetylation (dCas9-Gcn5). When expressing the dCas9-Gcn5 fusion protein, efficient transportation to the nucleus and sufficient expression in yeast cells are important. Therefore, we attempted to construct a dCas9-Gcn5 expression system that meets these two criteria. First, we constructed expression plasmids to examine whether the dCas9-Gcn5-EGFP fusion protein was localized to the nucleus (Figure 1A and Figure S1A). A multicopy plasmid, pWGP3 [33], or centromeric plasmid, p414 GPD [34], was used as the backbone vector for constitutive expression under the *GPD* promoter.

**Figure 2:**
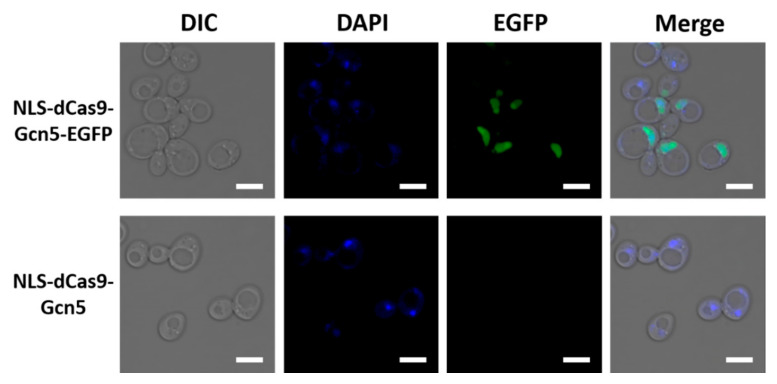
Confocal microscopy fluorescence images of *S. cerevisiae* expressing dCas9-Gcn5 fusion. Fluorescence images of *S. cerevisiae* UCC3505 transformed with p414 GPD_NLS-das9-Gcn5-EGFP and p414 GPD_NLS-dCas9-Gcn5. DIC, differential interference contrast image; DAPI, DAPI fluorescence image; EGFP, enhanced GFP fluorescence image; Merge, the merged images of DIC, DAPI, and EGFP. Scale bar, 5 µm.

The nuclear localization of the fusion protein was observed using confocal microscopy after staining with DAPI to visualize the nucleus (Figure 2 and Figure S2). The nuclear localization of dCas9-Gcn5-EGFP fusion protein was detected in both plasmids. We compared the number of cells expressing the dCas9-Gcn5-EGFP fusion protein based on EGFP fluorescence between pWGP3 and p414 GPD. EGFP fluorescence was observed in 41% or 19% of the cells when p414 GPD or pWGP3 was used as a vector, respectively (Figure S3). This result indicates that the dCas9-Gcn5-EGFP fusion protein was expressed in more cells when p414 GPD was used as a vector. Therefore, we used p414 GPD_NLS-dCas9-Gcn5 for subsequent experiments.

### 3.2. Evaluation of Chromatin Loosening by Cell Growth Assay

**Figure 3:**
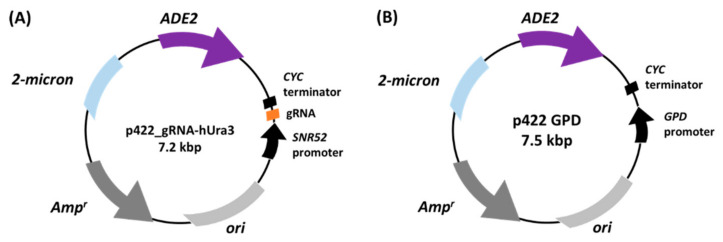
Construction of plasmids for producing gRNA. The gRNA was constitutively produced under the control of *SNR52* promoter by introducing multicopy plasmid p422_gRNA-hURA3 (**A**). For empty vector, p422 GPD (**B**) was constructed. gRNA, guide RNA.

We established an expression system for the dCas9-Gcn5 fusion protein, which is efficiently transported to the nucleus and produced in many yeast cells. In addition to dCas9-Gcn5, a gRNA recognizing a target locus in the heterochromatic region was expressed to promote chromatin loosening in the target locus. To detect chromatin loosening based on phenotypic alteration, we focused on *S. cerevisiae* UCC3505 [40]. This strain has a *ura3-52* mutation in the intrinsic *URA3* locus and *URA3* in the subtelomere region, which is known as the heterochromatic region. Therefore, although heterochromatic *URA3* is inactivated, the transition to the euchromatic state by chromatin loosening allows cells to grow on uracil-deficient medium. To induce chromatin loosening in the heterochromatic *URA3*, we expressed the dCas9-Gcn5 fusion protein and gRNA recognizing the upstream region of heterochromatic *URA3* in UCC3505 and monitored cell growth in the uracil-deficient medium. Yeast strains expressing the dCas9-Gcn5 fusion protein and gRNA were created by introducing p414GPD-NLS-dCas9-Gcn5 and p422_gRNA-hUra3 (Figure 3A) into UCC3505. As controls, empty plasmids p414 GPD and/or p422 GPD (Figure 3B) were introduced into UCC3505. Furthermore, to demonstrate that the chromatin loosening by the expression of dCas9-Gcn5 is due to a change in histone acetylation, the dCas9-Gcn5 was replaced by the dCas9-Gcn5^KQL126–128AAA^ mutant that loses histone acetyltransferase (HAT) activity [35,41,42]. In addition to these controls, an *ada2*Δ background was employed to examine whether the chromatin loosening depended on the formation of Spt-Ada-Gcn5-acetyltransferase (SAGA) complex or ADA complex and the resulting nucleosomal histone acetyltransferase activity. Yeast Gcn5 was reported to act on free histones alone but acetylate nucleosomal histones in these complexes via structural activation induced by interaction with Ada2 [28,42,43,44]. In the *ada2*Δ strain, Gcn5 cannot interact with any components of these native complexes [41]. Finally, as a positive control for the cell growth assay, the UCC3505 *URA3*-recovered strain, in which the *ura3-52* mutation was recovered, was transformed with p414 GPD and p422 GPD. 

**Figure 4:**
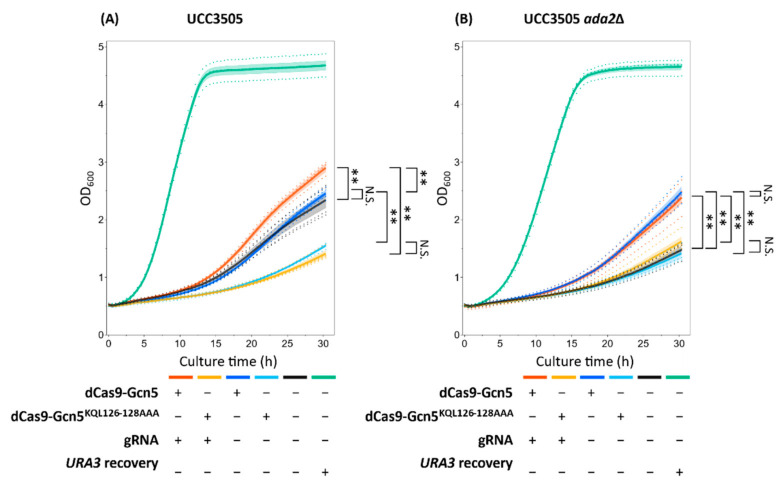
Cell growth in uracil-deficient medium. (**A**) Derivatives of UCC3505 strain, (**B**) derivatives of UCC3505 strain with deletion of *ADA2*. Yeast cells were precultured in SDC + 5 × U liquid medium for 48 h after transformation. The harvested cells were diluted to 0.2 of OD_600_ in SDC liquid medium. Thereafter, cell growth was monitored by measuring absorbance at 600 nm with the plate reader. Bands represent the SEM of three or four independent experiments and points represent each experimental data. Orange, the strain expressing dCas9-Gcn5 and gRNA; yellow, the strain expressing dCas9-Gcn5^KQL126–128AAA^ and gRNA; blue, the strain expressing dCas9-Gcn5 without gRNA; light blue, the strain expressing dCas9-Gcn5^KQL126–128AAA^ without gRNA; black, the strain harboring empty plasmids; green, the *URA3*-recovered strain harboring empty plasmids. A two-tailed Student’s test was used to assess the statistical significance, and the Holm method was used to control the family-wise error rates; ** *p* < 0.01, N.S. not significant (*p* > 0.05).

The growth of yeast transformants was monitored in uracil-deficient medium. In the presence of *ADA2*, the expression of dCas9-Gcn5 fusion protein and gRNA induced significantly greater growth than the expression of dCas9-Gcn5 without gRNA or no expression (empty plasmids) in terms of endpoints cell growth (Figure 4A) and maximum specific growth rate (Figure S4A). On the other hand, the expression of dCas9-Gcn5 without gRNA did not induce significantly greater growth than no expression (empty plasmids). Then, the strain expressing dCas9-Gcn5 and gRNA was subjected to plasmid loss and its growth was also monitored in uracil-deficient medium. As a result, its cell growth became slower (Figure S5). These results indicate that the gene expression was induced in the heterochromatic *URA3* by the expressed dCas9-Gcn5 in the gRNA-dependent manner. Furthermore, in the *ADA2* background, the expression of dCas9-Gcn5^KQL126–128AAA^ whose HAT activity is inactive [35,41,42] induced slower growth than their active counterparts (Figure 4A and Figure S4A). These results suggest the induced expression of *URA3* was caused by the HAT activity of dCas9-Gcn5 fusion protein.

Furthermore, the growth dependence of the strain expressing dCas9-Gcn5 fusion protein on gRNA expression was abolished in the *ada2*Δ background (Figure 4B and Figure S4B). Thus, gRNA-dependent expression of the heterochromatic *URA3* by dCas9-Gcn5 required the existence of Ada2. From these phenotypic alterations, the association between inducing heterochromatic *URA3* by dCas9-Gcn5 fusion protein with gRNA and heterochromatin loosening by Ada2-dependent Gcn5 nucleosomal histone acetylation was indirectly indicated in the targeted heterochromatic region.

On the other hand, in the *ada2*Δ background, dCas9-Gcn5 induced significantly greater growth than no expression (empty plasmids) and the expression of catalytically inactive dCas9-Gcn5^KQL126–128AAA^ independent of the expression of gRNA (Figure 4B and Figure S4B). This result suggests Gcn5 HAT activity-dependent but Ada2-independent induction of gene expression occurred in heterochromatic *URA3* by dCas9-Gcn5 in the gRNA-independent manner.

### 3.3. Examination of Chromatin Loosening by FAIRE-qPCR

To validate the chromatin loosening indicated by phenotypic alterations, we further analyzed the chromatin condensation levels in the target heterochromatic region and a non-target euchromatic region using FAIRE-qPCR. FAIRE-qPCR is one of the methods that can evaluate chromatin condensation levels by collecting and quantifying euchromatic DNA [45,46].

**Figure 5:**
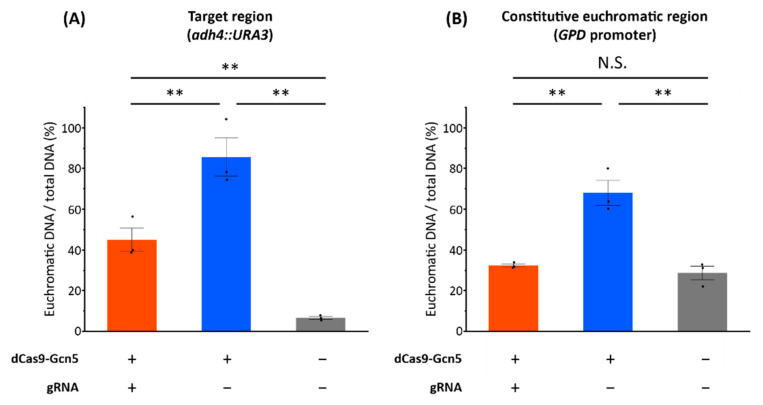
Evaluation of chromatin loosening by FAIRE-qPCR analysis. (**A**) FAIRE-qPCR results of the target region (heterochromatic *URA3*), (**B**) FAIRE-qPCR results of the constitutive euchromatic region (*GPD* promotor). dCas9-Gcn5 “+” or “−” indicates the cells harboring p414 GPD_ NLS-dCas9-Gcn5 or empty plasmid, respectively. gRNA “+” or “−” indicates the cells harboring p422_gRNA-hUra3 or empty plasmid, respectively. Chromatin loosening was evaluated by normalizing the amount of euchromatic DNA by the amount of total DNA. Error bars represent the SEM of three independent experiments and points represent each experimental data. Orange, the strain expressing dCas9-Gcn5 and gRNA; blue, the strain expressing dCas9-Gcn5 without gRNA; gray, the strain harboring empty plasmids. A two-tailed Student’s test was used to assess the statistical significance, and the Holm method was used to control the family-wise error rates; ** *p* < 0.01, N.S. not significant (*p* > 0.05).

Prior to FAIRE-qPCR analysis, the successive culture in liquid uracil-deficient medium and the subsequent isolation of single conies on uracil-deficient agar plates were performed to obtain the cells with enough chromatin loosening. The obtained single colonies expressing dCas9-Gcn5 fusion protein and gRNA showed improved growth phenotypes compared not only to the strains harboring empty plasmids but also to the *URA3*-recovered strains (Figure S6). The cell growth of the strain expressing dCas9-Gcn5 without gRNA was also improved as well as the strain expressing dCas9-Gcn5 and gRNA (Figure S6). These single colonies were subjected to FAIRE-qPCR. The chromatin in the target region was loosened by the expression of dCas9-Gcn5 with or without gRNA (Figure 5A). These results indicate that chromatin loosening was induced in the target region by dCas9-Gcn5.

On the other hand, the percentage of euchromatic DNA in the non-target euchromatic region (*GPD* promoter) did not differ between the strain expressing dCas9-Gcn5 and gRNA and the strain harboring empty plasmids (Figure 5B). However, this region of the strain expressing dCas9-Gcn5 without gRNA was also highly loosened (Figure 5B). These results suggest that chromatin loosening seemed to be nonspecifically induced in a non-target region without gRNA expression. 

## 4. Discussion

In this study, we designed a dCas9-Gcn5 fusion protein to introduce histone acetylation at a target locus in heterochromatic region. The cell growth assay (Figure 4 and Figure S4) and FAIRE-qPCR analysis (Figure 5) showed that the expressed dCas9-Gcn5 and gRNA played a functional role in loosening chromatin at the target heterochromatic locus through nucleosomal histone acetylation activity of Gcn5.

Growth phenotype in uracil-deficient medium, indicative of chromatin loosening, was most likely induced by histone acetylation via dCas9-Gcn5 fusion protein. The role of HAT activity in this process was verified by less growth of the strains expressing the fusion protein of dCas9 and catalytically inactive Gcn5 (Figure 4 and Figure S4). However, the introduction of the catalytically inactive Gcn5 reduced the cell growth in comparison to the control strain harboring empty plasmid (Figure 4A and Figure S4A). Therefore, it is impossible to separate the effects of HAT activity from other ones such as competitive inhibition of native Ada2 interaction. Nevertheless, this toxic effect of the Gcn5 ^KQL126–128AAA^ mutation was not significant in *ada2*Δ background strains (Figure 4B and Figure S4B), indicating that HAT activity and the resulting histone acetylation are required for chromatin loosening at least there. Therefore, it seems that there was an effect of histone acetylation in the improved growth of the strains expressing dCas9-Gcn5 fusion protein.

Moreover, the gRNA-dependent chromatin loosening in the target region by dCas9-Gcn5 fusion protein was observed (Figure 4) and it was most likely caused by well-known Ada2-dependent Gcn5 nucleosomal histone acetylation activity [28,41,42,43,44]. This could be supported by the significant growth difference between strains expressing dCas9-Gcn5 with and without gRNA only in the *ADA2* background strains (Figure 4 and Figure S4). The yeast SAGA complex consists of other proteins showing HAT activity [41], such as Taf1 [47] and Elp3 [48]. It also consists of other proteins promoting gene expression and sometimes affecting the chromatin loosening such as activator binding, TBP binding [49], and deubiquitination module components [50]. Thus, gRNA-dependent chromatin loosening with Ada2 can be also explained by their functions following the recruitment of the native SAGA complex to the target locus by the interaction between gRNA, dCas9-Gcn5, and Ada2. However, the non-significant difference in growth between the expression of catalytically inactive dCas9-Gcn5 with and without gRNA (Figure 4A and Figure S4A) suggests that these effects were minor compared to the nucleosomal histone acetylation activity of Gcn5. Therefore, dCas9-Gcn5 fusion protein was suggested to induce chromatin loosening in the heterochromatic region by nucleosomal histone acetylation following recruitment of dCas9-Gcn5 to the target locus by gRNA and enhancement of Gcn5 activity by Ada2.

On the other hand, gRNA- and Ada2-independent chromatin loosening was also induced by HAT activity of dCas9-Gcn5 fusion protein. Even in the absence of gRNA, cell growth was also improved by HAT activity of dCas9-Gcn5 in the *ada2*Δ background strain (Figure 4B and Figure S4B). After the single colony isolation, chromatin loosening was also detected in the strain expressing dCas9-Gcn5 without gRNA (Figure S6 and Figure 5A). These results may be explained by the acetylation of free histones by Ada2-independent intrinsic Gcn5 HAT activity [35,43,48,51,52] and their global loosening effects on the chromatin condensation states. This suggestion is supported by the high level of chromatin loosening in the non-target region in the absence of gRNA (Figure 5B). Therefore, in the case of no expression of gRNA, dCas9-Gcn5 fusion protein could cause chromatin loosening globally by its HAT activity.

By using our system, we could obtain yeast strains showing the loosened chromatin in the target heterochromatic locus after the single colony isolation of transformants in uracil-deficient medium (Figure 5A). The dCas9-Gcn5 together with gRNA enables the expression of the target heterochromatic *URA3* with the comparable level to that of the *URA3*-recovered positive control (Figure S6), and chromatin loosening in the target regions (*adh4::URA3*) with the percentage of euchromatic DNA comparable to the constitutive euchromatic region (*GPD* promoter) (Figure 5). Thus, our method of chromatin loosening in heterochromatic loci by dCas9-Gcn5 with gRNA through nucleosomal histone acetylation activity is a promising method to induce euchromatic state in a target genomic locus.

However, the cell growth promoted by the induced expression of the target heterochromatic *URA3* after transformation with dCas9-Gcn5 expressing plasmids was smaller than that of *URA3*-recovred cells (Figure 4 and Figure S4). Therefore, there is room for improvement in the efficiency of chromatin loosening. Growth selection in uracil-deficient medium and single colony isolation after transformation gave the strains with improved growth (Figure S6), making it possible to obtain the strains with high-level chromatin loosening. However, this process might increase the off-target chromatin loosening such as highly loosened *GPD* promoter region in the expression of dCas9-Gcn5 without gRNA (Figure S6 and Figure 5B). This effect could be suppressed by gRNA expression at least in the *GPD* promoter region (Figure 5B). Therefore, optimizing expression levels of dCas9-Gcn5 and gRNA could provide the efficient chromatin loosening with globally low-level off-target effects. Previous studies have shown that chromatin is loosened under stress conditions in *S. cerevisiae* [53]. The effect of preculturing in uracil-deficient medium on the elevated level of euchromatin by dCas9-Gcn5 (Figure S6 and Figure 5) may be explained by natively chromatin loosening triggered by stress conditions, such as uracil-deficiency and long-time incubation in non-nutrient rich SD medium. Such loosening would enable dCas9-Gcn5 to access heterochromatin more easily or enhances chromatin loosening after the nucleosomal histone acetylation. Therefore, to improve the chromatin loosening efficiency with keeping low-level off-target effects in global genomic regions, inducible promoters [54] or optogenetic gene expression systems [55] could be employed in our system, enabling temporary HATs activity at the specific cellular state suitable for more efficient chromatin loosening. In addition to Gcn5, the use or fusion of an enzyme that can acetylate other histone regions distinct from Gcn5, such as Esa1 [27,56], may improve the efficiency of chromatin loosening. Moreover, we found a critical role of Ada2 in chromatin loosening by the expression of Gcn5 and gRNA (Figure 4 and Figure S4). Therefore, the additional fusion of proteins to dCas9-Gcn5, such as Ada2 or other SAGA complex components, [28,42,43,44,49,50] are expected to have a positive effect on chromatin loosening activity.

In this study, the target region is a subtelomeric region, which is known to be a facultative heterochromatic region [57]. Therefore, it can be inferred that this method works efficiently on facultative heterochromatin. Since chromatin loosening by acetylation can work anywhere, our system can be expected for use to loosen chromatin structure in constitutive regions. In future studies, the detailed processes for chromatin loosening and locus specificity would be examined through the experiments such as measurement of histone acetylation level and FAIRE-qPCR analysis in different cellular states and non-target regions to investigate applicability of our system in various applications.

Although previous studies have fused histone-modifying enzymes different from Gcn5 to CRISPR/dCas9 to regulate the transcription levels of specific genes, these studies have not examined chromatin structure [20,21,22]. In addition, they did not examine the artificial regulation of gene expression in the heterochromatic regions. In this study, we achieved chromatin loosening in the heterochromatic region by expressing dCas9-Gcn5 and gRNAs. Chromatin condensation was reverted when this expression system was removed (Figure S4). Therefore, this system could be utilized to regulate chromatin condensation levels promptly by combining it with another system to induce chromatin condensation. This suggests that our technology has potential applicability in many fields. Since *S. cerevisiae*, the organism used in this study, has been utilized in various fields such as food fermentation and biofuel production [58], this system would be useful in improving the fermentation efficiency of yeast cells. Furthermore, the system makes it possible to suppress chromosomal abnormalities and contributes to the development of the treatment of diseases caused by chromosomal aberrations. Changes in chromatin structure are known to induce cell differentiation [13]. Hence, using this system, it would be possible to control cell conditions, such as differentiation and dedifferentiation, leading to the treatment of cancer and neurological diseases.

In conclusion, we developed an artificial technology to induce chromatin loosening in yeast. This system is a useful tool that overcomes the disadvantages of conventional chemical-catalyst approaches. To the best of our knowledge, this is the first study to induce chromatin loosening in a target heterochromatic locus by nucleosomal histone acetylation through the expression of dCas9-Gcn5 fusion and gRNA. In the future, our system can be utilized for a wide range of biological applications.

## Data Availability

The data underlying this article are available in the article and online Supplementary Materials.

## Supplementary Materials

The following are available online at https://www.mdpi.com/article/10.3390/biom12081138/s1, Figure S1: Construction of multi-copy plasmids for producing fusion protein of dCas9, Gcn5, and EGFP; Figure S2: Fluorescence images by confocal microscopy of *S. cerevisiae* UCC3505 harboring pWGP3_NLS-das9-Gcn5-EGFP or pWGP3_NLS-dCas9-Gcn5; Figure S3: Fluorescence images by confocal microscopy of *S. cerevisiae* W303-1A harboring p414 GPD_NLS-dCas9-Gcn5-EGFP or pWGP3_NLS-dCas9-Gcn5-EGFP; Figure S4: Maximum specific growth rate of cells in uracil-deficient medium; Figure S5: Cell growth in uracil-deficient medium after single colony isolation; Figure S6: Cell growth in uracil-deficient medium after single colony isolation; Table S1: List of yeast strains used in this study; Table S2: List of primers used in this study; Table S3: List of plasmids used in this study; and Table S4: Sequences of NLS and linker.
