# Supplementary material for: Development of Artificial System to Induce Chromatin Loosening in Saccharomyces cerevisiae
Source: Biomolecules. 2022 Aug 18;12(8):1138. doi: 10.3390/biom12081138 (PMC9406041; doi:10.3390/biom12081138)
Supplement: Supplementary file 1 [file biomolecules-12-01138-s001.zip › Supplementary Figures (Yamamoto et al).pdf]

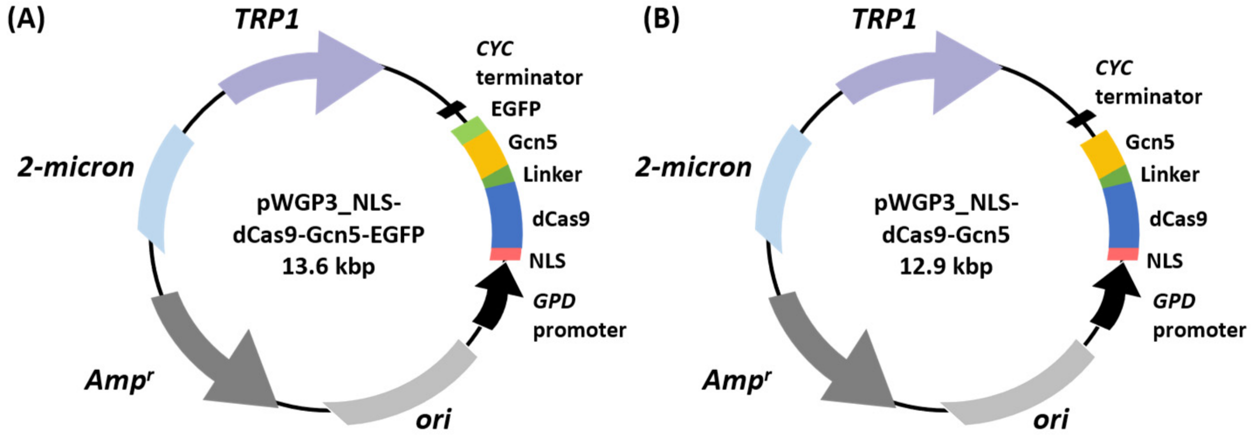

Figure S1. Construction of multi-copy plasmids for producing fusion protein of dCas9, Gcn5, and EGFP. NLS-dCas9-Gcn5-EGFP or NLS-dCas9-Gcn5 was constitutively produced under the control of the *GPD* promoter by introducing the multicopy plasmid pWGP3_NLS-dCas9-Gcn5-EGFP (A) or pWGP3_NLS-dCas9-Gcn5 (B), respectively.

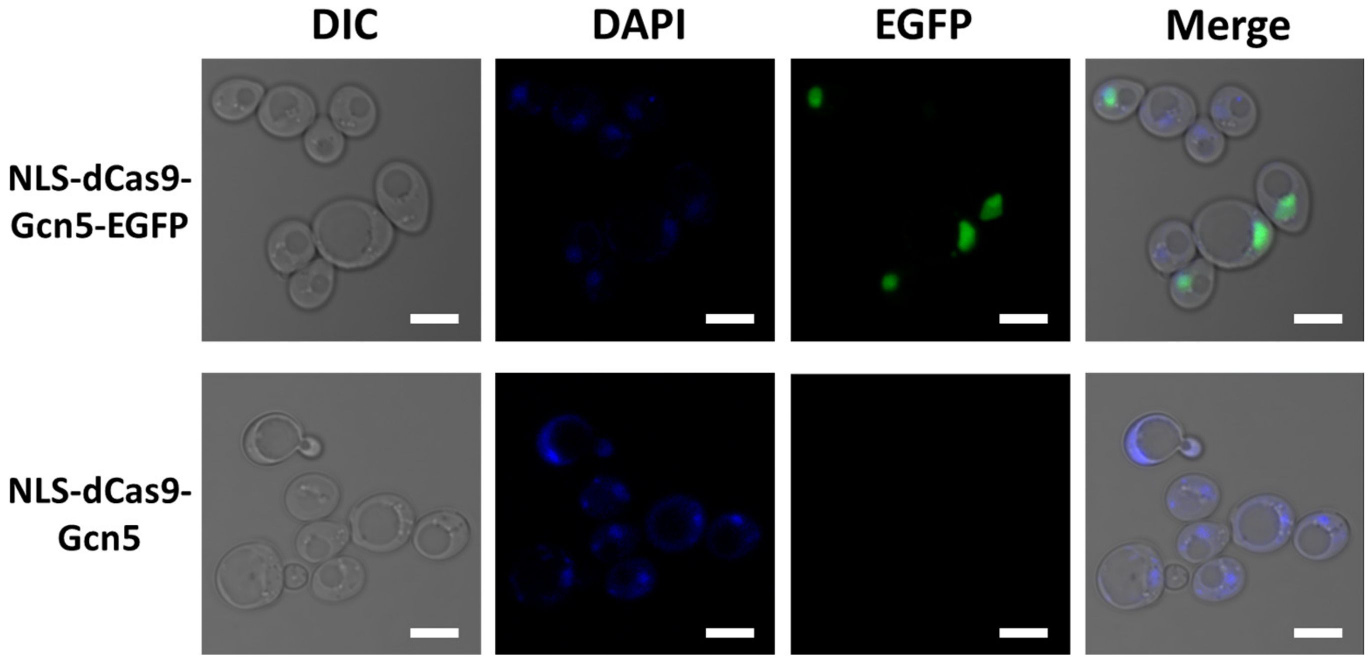

Figure S2. Fluorescence images by confocal microscopy of *S. cerevisiae* UCC3505 harboring pWGP3_NLS-das9-Gcn5-EGFP or pWGP3_NLS-dCas9-Gcn5. DIC, differential interference contrast image; DAPI, DAPI fluorescence image; EGFP, enhanced GFP fluorescence image; Merge, the merged images of DIC, DAPI, and EGFP. Scale bar, 5 µm.

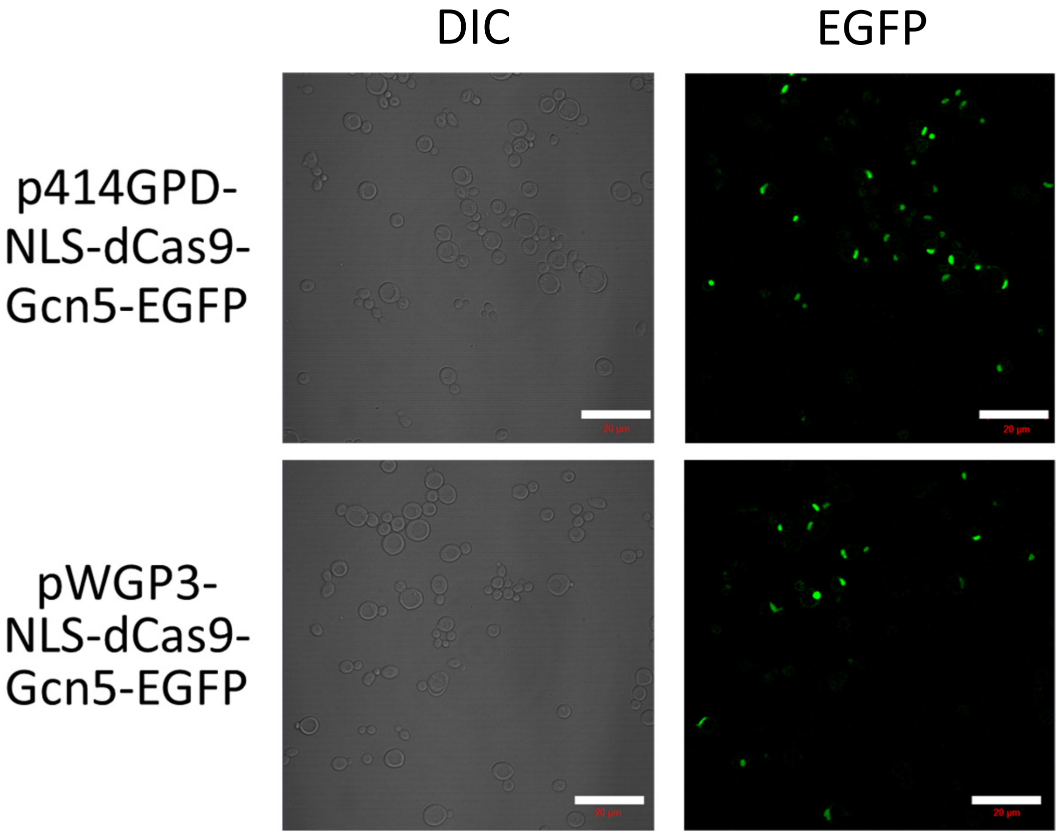

Figure S3. Fluorescence images by confocal microscopy of *S. cerevisiae* W303-1A harboring p414 GPD_NLS-dCas9-Gcn5-EGFP or pWGP3_NLS-dCas9-Gcn5-EGFP. DIC, differential interference contrast image; EGFP, enhanced GFP fluorescence image. Scale bar, 20 μm.

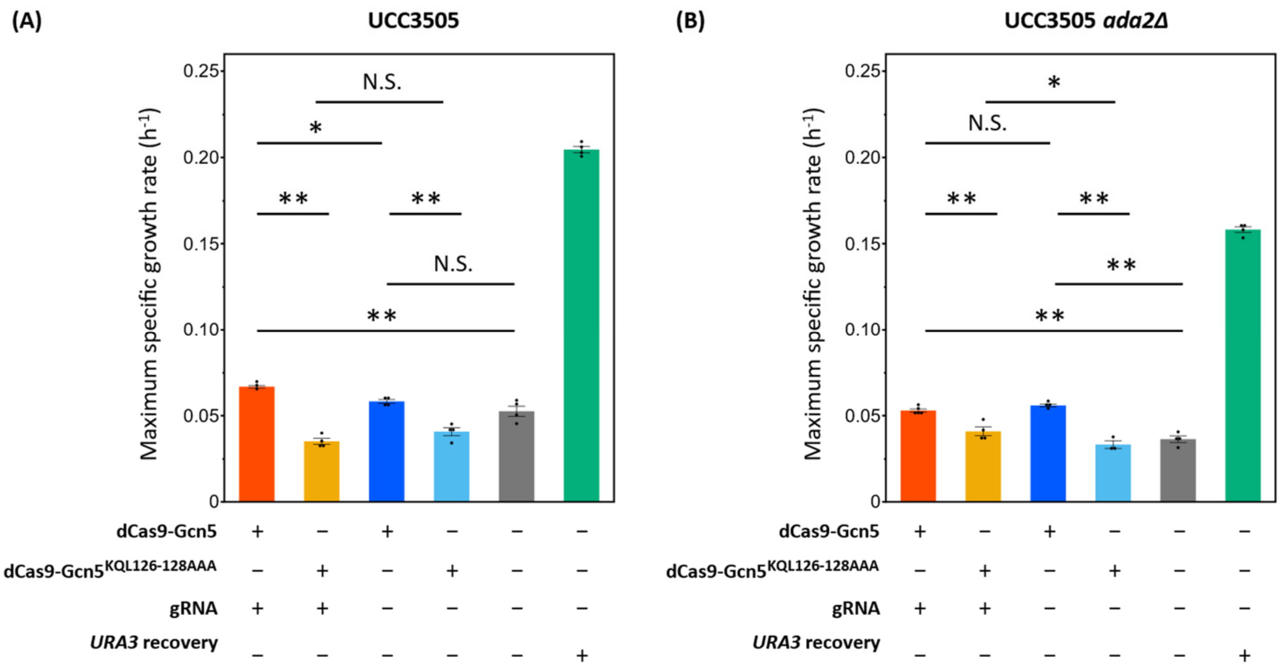

Figure S4. Maximum specific growth rate of cells in uracil-deficient medium (A) derivatives of UCC3505 strain, (B) derivatives of UCC3505 strain with deletion of *ADA2.* Yeast cells were precultured in SDC + 5 × U liquid medium for 48 h after transformation. The harvested cells were diluted to 0.2 of OD_{600} in SDC liquid medium. Thereafter, cell growth was monitored by measuring OD_{600} with the plate reader. Maximum specific growth rate was calculated by using Curveball 0.2.16 [39]. Error bars represent the SEM of three to five independent experiments. Points represent each experimental data. From left to right: the strain expressing dCas9-Gcn5 and gRNA; the strain expressing dCas9-Gcn5$^{KQL126-128AAA}$ and gRNA; the strain expressing dCas9-Gcn5 without gRNA; the strain expressing dCas9-Gcn5$^{KQL126-128AAA}$ without gRNA; the strain harboring empty plasmids; the *URA3*-recovered strain harboring empty plasmids. A two-tailed Student's test was used to assess the statistical significance and the Holm method was used to control the family-wise error rates;**$p<0.01$, *$p<0.05$, N.S. not significant.

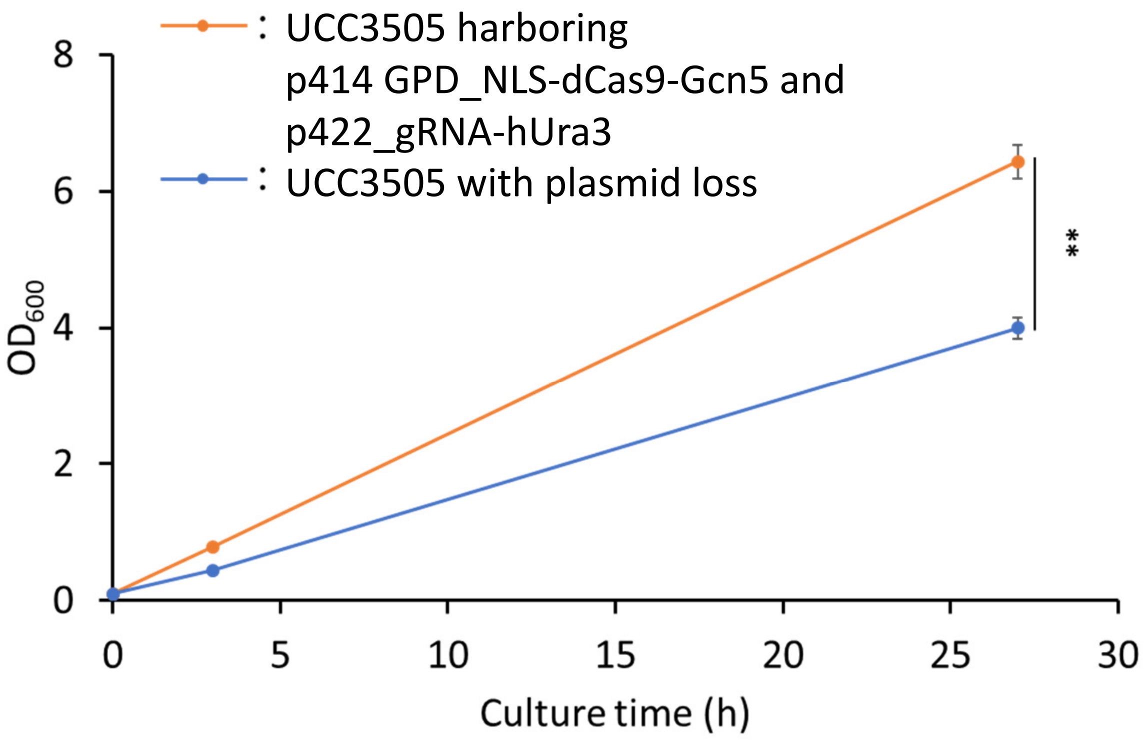

Figure S5. Cell growth in uracil-deficient medium. Yeast cells were precultured in SDC+U or SDC+AWU medium overnight. The harvested cells were diluted to 0.1 of OD_{600} in uracil-deficient medium, then cell growth was evaluated by measuring OD_{600}. UCC3505 harboring p414 GPD_NLS-dCas9-Gcn5 and p422_gRNA-hUra3 was cultured in SDC liquid medium, and UCC3505 with plasmid loss was cultured in SDC + AW liquid medium. Error bars represent the SEM of three independent experiments. Orange, UCC3505 harboring p414 GPD_NLS-dCas9-Gcn5 and p422_gRNA-hUra3; Blue, UCC3505 with plasmid loss. A two-tailed Student's test was used to assess the statistical significance; **$p<0.01$.

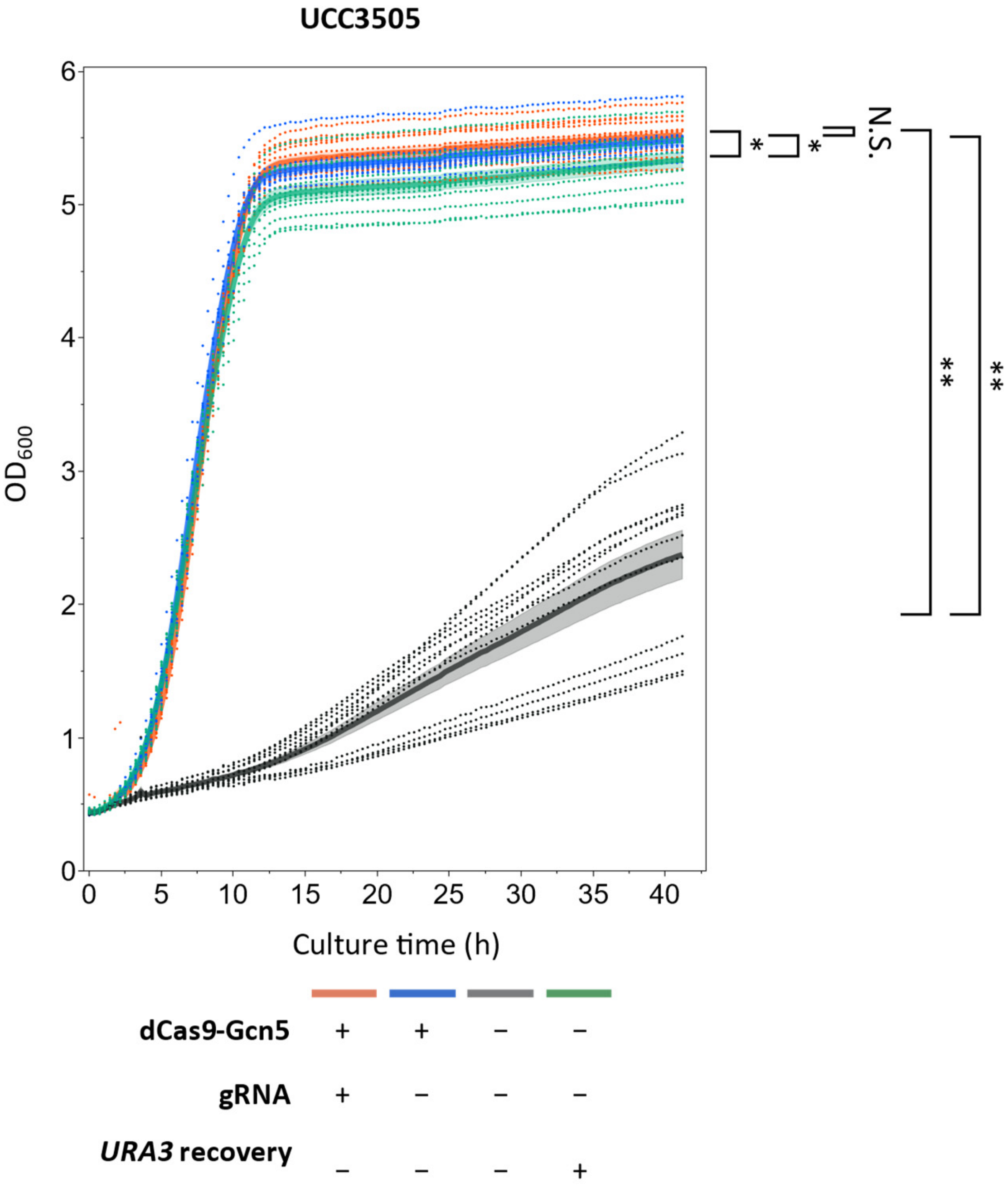

Figure S6. Cell growth in uracil-deficient medium after single colony isolation. Yeast cells Isolated as single colonies were precultured in SDC + U liquid medium overnight. The harvested cells were diluted to 0.2 of OD_{600} in SDC liquid medium. Thereafter, cell growth was monitored by measuring OD_{600} with the plate reader. Bands represent the SEM of three independent single colonies and four independent experiments. Points represent each experimental data. Orange, the strain expressing dCas9-Gcn5 and gRNA; Blue, the strain expressing dCas9-Gcn5 without gRNA; Gray, the strain harboring empty plasmids; Green, the *URA3*-recovered strain harboring empty plasmid. A two-tailed Student's test was used to assess the statistical significance, and the Holm method was used to control the family-wise error rates; *$p < 0.05$, **$p<0.01$., N.S. not significant.
